# Supplementary material for: Antimicrobial resistance, equity and justice in low- and middle-income countries: an intersectional critical interpretive synthesis
Source: Nat Commun. 2025 Oct 13;16:9078. doi: 10.1038/s41467-025-64137-z (PMC12518637; doi:10.1038/s41467-025-64137-z)
Supplement: Supplementary file 1 — Supplementary Information [file 41467_2025_64137_MOESM1_ESM.pdf]

Supplementary information

Table S1: Example database search terms (Scopus)

Domain	Search Terms
AMR domain	TITLE-ABS-KEY(AMR OR "antimicrobial resistan*" OR "drug-resistan*" OR "multidrug resistan*" OR MDR OR "multiple drug resistan*" OR "antibiotic resistan*" OR ABR OR XDR OR "extensively drug resistan*" OR "antimicrobial access" OR "antimicrobial use" OR "antimicrobial stewardship" OR “antibiotic use” OR “antibiotic access”)
Equity domain	TITLE-ABS-KEY(gender* OR “non-binary” OR intersectional* OR feminis* OR sexis* OR masculinit* OR femininit* OR equit* OR inequit* OR equalit* OR inequalit* OR refugee* OR “displaced person*” OR “asylum seeker*” OR disabilit* OR ethnicity OR “ethnic minorit*” OR “racial minorit*” OR “sociocultural factor*” OR “socioeconomic factor*” OR “social determinant*” OR “socioeconomic determinant*” OR “socio-economic factor” OR “socio-economic determinant” OR “interpersonal relation*” OR “social norm*” OR “societal norm*” OR “gender norm*” OR “gender relations” OR “women’s mobility” OR “gender transformative” OR transgender OR “two spirit*” OR sexuality OR “sex worker*” OR “gender-based violence” OR “intimate partner violence” OR “health justice” OR “environmental justice” OR “gender justice” OR “social justice” OR poverty OR “marginalised communities”)
LMIC domain	TITLE-ABS-KEY (Africa or Asia or Caribbean or "South America" or "Latin America" or "Central America" or "middle east")
	TITLE-ABS-KEY(Afghanistan OR Angola OR Bangladesh OR Benin OR "Burkina Faso" OR Burundi OR Cambodia OR "Central African Republic" OR Chad OR

	Comoros OR Congo OR Djibouti OR Eritrea OR Ethiopia OR Gambia OR Guinea OR "Guinea-Bissau" OR Haiti OR Kiribati OR Lao OR Laos)
	TITLE-ABS-KEY (Lesotho OR Liberia OR Madagascar OR Malawi OR Mali OR Mauritania OR Micronesia OR Mozambique OR Myanmar OR Nepal OR Niger OR Rwanda OR "São Tomé and Principe" OR Senegal OR "Sierra Leone" OR "Solomon Islands" OR Somalia OR Sudan OR Tanzania OR "Timor-Leste" OR Togo OR Tuvalu OR Uganda OR Yemen OR Zambia)
	TITLE-ABS-KEY ("North Korea" OR "Democratic People's Republic of Korea" OR "Syrian Arab Republic" OR Syria OR Algeria OR Bhutan OR Bolivia OR "Cabo Verde" OR Cameroon OR Congo OR "Côte d'Ivoire" OR Egypt OR Eswatini OR Ghana OR Honduras OR India OR Iran OR Jordan OR Kenya OR "Kyrgyz republic" OR Kyrgyzstan OR Lebanon)
	TITLE-ABS-KEY (Micronesia OR Mongolia OR Morocco OR Nicaragua OR Nigeria OR Pakistan OR "Papua New Guinea" OR Philippines OR Samoa OR "Sri Lanka" OR Tajikistan OR Tokelau OR Tunisia OR Ukraine OR Uzbekistan OR Vanuatu OR "Viet Nam" OR vietnam OR Zimbabwe OR Albania OR Argentina OR Armenia OR Azerbaijan OR Belarus OR Belize OR Bosnia OR Herzegovina OR Botswana OR Brazil OR China OR Colombia OR Costa Rica OR Cuba OR Dominica OR "Dominican Republic")
	TITLE-ABS-KEY (Ecuador OR "El Salvador" OR "Equatorial Guinea" OR Fiji OR Gabon OR Georgia OR Grenada OR Guatemala OR Indonesia OR Iraq OR Jamaica OR Kazakhstan OR Kosovo OR Libya OR Malaysia OR Maldives OR "Marshall Islands" OR Mauritius OR Mexico OR Moldova OR Montenegro OR Namibia OR Niue OR "North Macedonia" OR Palau OR Paraguay OR Peru OR "Saint Helena" OR "Saint Lucia" OR "Saint Vincent and the Grenadines" OR Serbia OR "South Africa" OR Suriname OR Thailand OR Tonga OR Türkiye OR

	Turkey OR Turkmenistan OR "Wallis and Futuna" OR "West Bank" OR "Gaza Strip" OR Palestine)
	TITLE-ABS-KEY ((developing or “less* developed” or “under developed” or underdeveloped or “middle income” or “low* income” or underserved or “under served” or deprived or poor*) W/1 (countr* or nation? or population? or world))
	TITLE-ABS-KEY ((developing or "less* developed" or "under developed" or underdeveloped or "middle income" or "low* income") W/1 (economy or economies))
	TITLE-ABS-KEY (low* W/1 (gdp or gnp or "gross domestic" or "gross national"))
	TITLE-ABS-KEY (low W/3 middle W/3 countr*)
	TITLE-ABS-KEY (lmic or lmics or "third world" or "lami countr*")
	TITLE-ABS-KEY ("transitional countr*")
	TITLE-ABS-KEY (("high burden" or high-burden or countdown) W/1 countr*)

Table S2: Inclusion and exclusion criteria

**Inclusion criteria**	**Exclusion criteria**
Provide insights into how social or structural determinants of health impact on susceptibility or exposure to infection, transmission routes for AMR, access to treatment or the impact of the disease	Articles that reported only on burden of disease
Articles that focus on Low- or Middle-income settings (as defined by (179))	Articles that focus on high income countries

Articles that have been published within the last ten years (2014 - 2024) in order to synthesise current evidence and thereby provide evidence summaries that are based on up-to-date and relevant research	Articles published before 2014
Peer reviewed journal articles reporting on results of primary research or secondary data analysis	Commentaries, review articles

Table S3: Data extraction template

**Article data**						**Study data**			**Themes**												
**Author**	Year	Title	Abstract	DOI	Source	Country	Disease/drug	Population	Susceptibility to infection	Exposure to infection and antimicrobials	Access to diagnosis and healthcare	Self-medication with antimicrobials	Knowledge of AMR and antimicrobials	Prescription practices	Treatment completion/continuity	Experiences of care	Impacts (e.g. stigma)	Interventions	Intersectionality	Other findings	One Health domain

Table S4: Included articles

Citation	Countries	Infection/Drug	Susceptibility to infection	Exposure to infection and antimicrobials	Access to diagnosis and healthcare	Self-medication with antimicrobials	Knowledge of AMR and antimicrobials	Prescription practices	Treatment completion/ continuity	Experiences of care	Impacts (e.g. stigma)	One Health domain
Atterby, Clara, Kristina Osbjer, Viktoria Tepper, Elisabeth Rajala, Jorge Hernandez, Sokerya Seng, Davun Holl et al. "Carriage of carbapenemase-and extended-spectrum cephalosporinase-producing Escherichia coli and Klebsiella pneumoniae in humans and livestock in rural Cambodia; gender and age differences and detection of blaOXA-48 in humans." Zoonoses and public health 66, no. 6 (2019): 603-617.	Cambodia	carbapenemase-producing *Escherichia coli/Klebsiella pneumoniae*		x								HH, AH, EE
Bannister-Tyrrell, M., Gryseels, C., Sokha, S., Dara, L., Sereiboth, N., James, N., Thavrin, B., Ly, P., Ty, K.S., Grietens, K.P. and Sovannaroth, S., 2019. Forest goers and multidrug-resistant malaria in Cambodia: an ethnographic study. The American journal of tropical medicine and hygiene, 100(5), p.1170.	Cambodia	Malaria	x	x	x							HH

Berendes, D., Knee, J., Sumner, T., Capone, D., Lai, A., Wood, A., Patel, S., Nalá, R., Cumming, O. and Brown, J., 2019. Gut carriage of antimicrobial resistance genes among young children in urban Maputo, Mozambique: Associations with enteric pathogen carriage and environmental risk factors. PloS one, 14(11), p.e0225464.	Mozambique	Multiple		x								HH, EH
Chanvatik, S., Kosiyaporn, H., Lekagul, A., Kaewkhankhaeng, W., Vongmongkol, V., Thunyahan, A. and Tangcharoensathien, V., 2019. Knowledge and use of antibiotics in Thailand: A 2017 national household survey. PloS one, 14(8), p.e0220990.	Thailand	NA					x					HH
Chowdhury, M., Stewart Williams, J., Wertheim, H., Khan, W.A., Matin, A. and Kinsman, J., 2019. Rural community perceptions of antibiotic access and understanding of antimicrobial resistance: qualitative evidence from the Health and Demographic Surveillance System site in Matlab, Bangladesh. Global Health Action, 12(sup1), p.1824383.	Bangladesh	NA			x							HH
Fleece, M.E., Nshama, R., Walongo, T., Kimathi, C., Gratz, J., McQuade, E.T.R., Liu, J., Pholwat, S., Mduma, E., Houpt, E.R. and Platts-Mills, J.A., 2019.	Tanzania	Antibiotic susceptibility profiles of Escherichia coli		x								HH

Longitudinal assessment of antibiotic resistance in fecal Escherichia coli in Tanzanian children. The American Journal of Tropical Medicine and Hygiene, 100(5), p.1110.												
Ha, T.V., Nguyen, A.M.T. and Nguyen, H.S.T., 2019. Public awareness about antibiotic use and resistance among residents in highland areas of Vietnam. BioMed research international, 2019(1), p.9398536.	Vietnam	Awareness of antibiotics use and resistance					x					HH
Hendriksen, R.S., Lukjancenko, O., Munk, P., Hjelmsø, M.H., Verani, J.R., Ng'eno, E., Bigogo, G., Kiplangat, S., Oumar, T., Bergmark, L. and Röder, T., 2019. Pathogen surveillance in the informal settlement, Kibera, Kenya, using a metagenomics approach. PLoS One, 14(10), p.e0222531.	Kenya	Pathogens and bacterial AMR genes obtained from urban sewage		x								HH
Jember, E., Feleke, A., Debie, A. and Asrade, G., 2019. Self-medication practices and associated factors among households at Gondar town, Northwest Ethiopia: a cross-sectional study. BMC research notes, 12(1), p.153.	Ethiopia	Self-medication practices				x						HH
Kadariya, J., Thapaliya, D., Bhatta, S., Mahatara, R.L., Bempah, S., Dhakal, N. and Smith, T.C., 2019. Multidrug-resistant Staphylococcus aureus	Nepal	Multidrug-resistant Staphylococcus aureus colonization		x								HH

colonization in healthy adults is more common in bhutanese refugees in nepal than those resettled in ohio. BioMed research international, 2019(1), p.5739247.												
Kadhim, Z.K. and Khudair, F.W., 2019. Outpatients' Attendants Knowledge Regarding Antibiotics Use and Antibiotics Resistance in Al-Najaf Al-Ashraf City Health Institutions. Indian Journal of Public Health Research & Development, 10(8).	Iraq	knowledge concerning antibiotics use and resistance					x					HH
Larson, A., Hartinger, S.M., Riveros, M., Salmon-Mulanovich, G., Hattendorf, J., Verastegui, H., Huaylinos, M.L. and Mäusezahl, D., 2019. Antibiotic-resistant Escherichia coli in drinking water samples from rural Andean households in Cajamarca, Peru. The American journal of tropical medicine and hygiene, 100(6), p.1363.	Peru	Resistant Escherichia coli in Drinking Water Samples	x	x							x	HH AH
Mate, I., Come, C.E., Gonçalves, M.P., Cliff, J. and Gudo, E.S., 2019. Knowledge, attitudes and practices regarding antibiotic use in Maputo City, Mozambique. PloS one, 14(8), p.e0221452.	Mozambique	NA				x	x	x				HH
Mussie, K.M., Yimer, S.A., Manyazewal, T. and Gradmann, C., 2019. Exploring local realities: Perceptions and experiences of healthcare workers on the	Ethiopia	DR-TB	x	x					x		x	HH

management and control of drug-resistant tuberculosis in Addis Ababa, Ethiopia. PLoS One, 14(11), p.e0224277.												
Pasaribu, L.R. and Puspandari, N., Risk Factors to Gonorrhoea among Female Sex Worker in Three Cities in Indonesia.	Indonesia	STIs	x	x								HH
Pearson, M. and Chandler, C., 2019. Knowing antimicrobial resistance in practice: a multi-country qualitative study with human and animal healthcare professionals. Global health action, 12(sup1), p.1599560.	Ethiopia, India, Nigeria, the Philippines, Sierra Leone and Vietnam	NA		x				x		x		HH and AH
Peterson, M.L., Gandhi, N.R., Clennon, J., Nelson, K.N., Morris, N., Ismail, N., Allana, S., Campbell, A., Brust, J.C., Auld, S.C. and Mathema, B., 2019. Extensively drug-resistant tuberculosis 'hotspots' and sociodemographic associations in Durban, South Africa. The International Journal of Tuberculosis and Lung Disease, 23(6), pp.720-727.	South Africa	XDR-TB		x	x							HH
Pham-Duc, P., Cook, M.A., Cong-Hong, H., Nguyen-Thuy, H., Padungtod, P., Nguyen-Thi, H. and Dang-Xuan, S., 2019. Knowledge, attitudes and practices of livestock and	Vietnam	NA				x						HH, AH

aquaculture producers regarding antimicrobial use and resistance in Vietnam. Plos one, 14(9), p.e0223115.												
Willis, L.D. and Chandler, C., 2019. Quick fix for care, productivity, hygiene and inequality: reframing the entrenched problem of antibiotic overuse. BMJ global health, 4(4).	Tanzania, Uganda	NA		x				x				HH, AH
Zhang, Y., Duan, L., Wang, B., Du, Y., Cagnetta, G., Huang, J., Blaney, L. and Yu, G., 2019. Wastewater-based epidemiology in Beijing, China: prevalence of antibiotic use in flu season and association of pharmaceuticals and personal care products with socioeconomic characteristics. Environment International, 125, pp.152-160.	China	NA		x								HH, EH
Al-Mustapha, A.I., Adetunji, V.O. and Heikinheimo, A., 2020. Risk perceptions of antibiotic usage and resistance: A cross-sectional survey of poultry farmers in Kwara State, Nigeria. Antibiotics, 9(7), p.378.	Nigeria	NA		x				x				AH, HH
Allel, K., García, P., Labarca, J., Munita, J.M., Rendic, M., de Resistencia Bacteriana, G.C. and Undurraga, E.A., 2020. Socioeconomic factors associated with antimicrobial resistance of Pseudomonas	Chile	P. aeruginosa, S. aureus, and E. coli		x	x							HH

aeruginosa, Staphylococcus aureus, and Escherichia coli in Chilean hospitals (2008–2017). Revista Panamericana de Salud Pública, 44, p.e30.												
Caudell, M.A., Dorado-Garcia, A., Eckford, S., Creese, C., Byarugaba, D.K., Afakye, K., Chansa-Kabali, T., Fasina, F.O., Kabali, E., Kiambi, S. and Kimani, T., 2020. Towards a bottom-up understanding of antimicrobial use and resistance on the farm: A knowledge, attitudes, and practices survey across livestock systems in five African countries. PloS one, 15(1), p.e0220274.	Ghana, Kenya, Tanzania, Zambia, Zimbabwe	Multiple			x			x				AH
Dowdy, D.W., Zwerling, A.A., Stennett, A., Searle, A., Dukhanin, V., Taylor, H.A. and Merritt, M.W., 2020. Measuring stigma to assess the social justice implications of health-related policy decisions: application to novel treatment regimens for multidrug-resistant tuberculosis. MDM Policy & Practice, 5(1), p.2381468320915239.	South Africa, Uganda	MDR-TB									x	HH
Egbule, O.S., Iweriebor, B.C. and Odum, E.I., 2020. Beta-Lactamase-producing Escherichia coli isolates recovered from pig handlers in retail shops and Abattoirs in selected localities in Southern	Nigeria	Escherichia coli		x								HH, AH

Nigeria: Implications for public health. Antibiotics, 10(1), p.9.												
Ekman, B., Paudel, P., Basnet, O., Kc, A. and Wrammert, J., 2020. Adherence to World Health Organisation guidelines for treatment of early onset neonatal sepsis in low-income settings; a cohort study in Nepal. BMC Infectious Diseases, 20(1), p.666.	Nepal	Neonatal sepsis						x				HH
Grant-McAuley, W., Fogel, J.M., Galai, N., Clarke, W., Breaud, A., Marzinke, M.A., Mbwambo, J., Likindikoki, S., Aboud, S., Donastorg, Y. and Perez, M., 2020. Antiretroviral drug use and HIV drug resistance in female sex workers in Tanzania and the Dominican Republic. Plos one, 15(10), p.e0240890.	Tanzania and Dominican Republic	HIV (ARV drug use and resistance)		x								HH
Haenssgen, M.J., Charoenboon, N., Xayavong, T. and Althaus, T., 2020. Precarity and clinical determinants of healthcare-seeking behaviour and antibiotic use in rural Laos and Thailand. BMJ Global Health, 5(12), p.e003779.	Thailand, Laos	The role of precarity as a contextual factor shaping healthcare-seeking behaviour	x	x	x							HH

Higuita-Gutiérrez, L.F., Roncancio Villamil, G.E. and Jiménez Quiceno, J.N., 2020. Knowledge, attitude, and practice regarding antibiotic use and resistance among medical students in Colombia: a cross-sectional descriptive study. BMC public health, 20(1), p.1861.	Colombia	Knowledge, attitude, and practice regarding antibiotic use and resistance				x	x	x				HH
Huque, R., Elsey, H., Fieroze, F., Hicks, J.P., Huque, S., Bhawmik, P., Walker, I. and Newell, J., 2020. “Death is a better option than being treated like this”: a prevalence survey and qualitative study of depression among multi-drug resistant tuberculosis in-patients. BMC Public Health, 20(1), p.848.	Bangladesh	Multi-drug resistant tuberculosis (MDR-TB)									x	HH
Louis, Frantz Jean, Jean Wysler Domercant, Caroline Ignacio, Sara Gianella, Guethina Galbaud, Maureen Leonard, Davey M. Smith, and Antoine Chaillon. "High prevalence of HIV-1 drug resistance and dynamics of transmission among high-risk populations in Port-Au-Prince, Haiti." JAIDS Journal of Acquired Immune Deficiency Syndromes 85, no. 4 (2020): 416-422.	Haiti	HIV		x								HH

Jimah, T. and Ogunseitan, O., 2022. Socio-demographic characteristics of the association between knowledge of antibiotic therapy and prudent use in Ghana.	Ghana	Knowledge, attitudes, and practices concerning antibiotics and antibiotic resistance					x					HH
Jimah, T., Fenny, A.P. and Ogunseitan, O.A., 2020. Antibiotics stewardship in Ghana: a cross-sectional study of public knowledge, attitudes, and practices among communities. *One Health Outlook*, *2*(1), p.12.	Ghana	Knowledge, attitudes, and practices concerning antibiotics and antibiotic resistance					x					HH
Kariuki, Samuel, Cecilia Mbae, Sandra Van Puyvelde, Robert Onsare, Susan Kavai, Celestine Wairimu, Ronald Ngetich, John Clemens, and Gordon Dougan. "High relatedness of invasive multi-drug resistant non-typhoidal Salmonella genotypes among patients and asymptomatic carriers in endemic informal settlements in Kenya." *PLOS Neglected Tropical Diseases* 14, no. 8 (2020): e0008440.	Kenya	Invasive multi-drug resistant non-typhoidal Salmonella (iNTS)	x	x								HH
Kristina, S.A., Wati, M.R., Prasetyo, S.D. and Fortwengel, G., 2020. Public knowledge and awareness towards antibiotics use in Yogyakarta: A cross sectional survey. *Pharmaceutical Sciences Asia*, *47*(2), pp.173-180.	Indonesia	NA				x	x	x	x			HH

Lindman, J., Djalo, M.A., Biai, A., Månsson, F., Esbjörnsson, J., Jansson, M., Medstrand, P., Norrgren, H. and SWEGUB CORE group N'Buna Babetida Biague Antonio Biai Ansu Camara Cidia da Silva Zacarias Jose Esbjörnsson Joakim Jansson Marianne Karlson Sara Lindman Jacob Medstrand Patrik Månsson Fredrik Norrgren Hans Sahin Gülsen Özkaya Wilhelmson Sten, 2020. The HIV care continuum and HIV-1 drug resistance among female sex workers: a key population in Guinea-Bissau. AIDS research and therapy, 17(1), p.33.	Guinea-Bissau	HIV and HIV-1 drug resistance		x	x							HH
Manderson, L., 2020. Prescribing, care and resistance: antibiotic use in urban South Africa. Humanities and Social Sciences Communications, 7(1), pp.1-10.	South Africa	NA	x	x	x	x		x				HH
Mishra, S., Suwannapong, N., Tipayamongkholgul, M. and Aimyong, N., 2020. Access to health service and social support related to self-medication. Journal of Nepal Health Research Council, 18(03), pp.500-505.	Nepal	NA				x						HH
Moffo, F., Mouiche, M.M.M., Kochivi, F.L., Dongmo, J.B., Djomgang, H.K., Tombe, P., Mbah, C.K., Mapiefou, N.P., Mingoas, J.P.K. and Awah-Ndukum, J., 2020. Knowledge,	Cameroon	NA				x	x					HH, AH

attitudes, practices and risk perception of rural poultry farmers in Cameroon to antimicrobial use and resistance. *Preventive veterinary medicine*, *182*, p.105087.												
Moktan, D. and Shehnaz, S.I., 2020. Factors driving self-medication with antimicrobials in Karaikal, Puducherry, India. *Journal of Pharmacology and Pharmacotherapeutics*, *11*(2), pp.64-71.	India	NA				x		x				HH
Mpagama, S.G., Ezekiel, M.J., Mbelele, P.M., Chongolo, A.M., Kibiki, G.S., de Guex, K.P. and Heysell, S.K., 2020. Gridlock from diagnosis to treatment of multidrug resistant tuberculosis (MDR-TB) in Tanzania: patients' perspectives from a focus group discussion. BMC Public Health, 20(1), p.1667.	Tanzania	MDR-TB			x				x	x	x	HH
Oga-Omenka, C., Bada, F., Agbaje, A., Dakum, P., Menzies, D. and Zarowsky, C., 2020. Ease and equity of access to free DR-TB services in Nigeria-a qualitative analysis of policies, structures and processes. International Journal for Equity in Health, 19(1), p.221.	Nigeria	DR-TB			x				x	x	x	HH
Oga-Omenka, C., Boffa, J., Kuye, J., Dakum, P., Menzies, D. and Zarowsky, C., 2020. Understanding the gaps in DR-TB	Nigeria	Drug resistant TB			x				x	x	x	HH

care cascade in Nigeria: a sequential mixed-method study. *Journal of Clinical Tuberculosis and Other Mycobacterial Diseases*, *21*, p.100193.												
Ramay, B.M., Caudell, M.A., Cordón-Rosales, C., Archila, L.D., Palmer, G.H., Jarquin, C., Moreno, P., McCracken, J.P., Rosenkrantz, L., Amram, O. and Omulo, S., 2020. Antibiotic use and hygiene interact to influence the distribution of antimicrobial-resistant bacteria in low-income communities in Guatemala. Scientific reports, 10(1), p.13767.	Guatemala	NA		x	x							HH
Rivillas-García, J.C., Sanchez, S.M. and Rivera-Montero, D., 2020. Social inequalities related to antimicrobial resistance in N. gonorrhoeae in ColombiaDesigualdades sociais relacionadas à resistência antimicrobiana de N. gonorrhoeae na Colômbia. Revista Panamericana de Salud Publica= Pan American Journal of Public Health, 44, pp.e49-e49.	Colombia	Gonorrhoeae			x		x					HH
Simon, B. and Kazaura, M., 2020. Prevalence and factors associated with parents self-medicating under-fives with antibiotics in Bagamoyo District Council, Tanzania: a cross-sectional study. Patient	Tanzania	NA			x							HH

preference and adherence, pp.1445-1453.												
Steenberg, B., 2020. HIV-positive Mozambican migrants in South Africa: loneliness, secrecy and disclosure. Culture, health & sexuality, 22(1), pp.48-63.	South Africa	HIV			x						x	HH
Wang, Y., McNeil, E.B., Huang, Z., Chen, L., Lu, X., Wang, C., Chen, H. and Chongsuvivatwong, V., 2020. Household financial burden among multidrug-resistant tuberculosis patients in Guizhou province, China: a cross-sectional study. Medicine, 99(28), p.e21023.	China	MDR-TB									x	HH
Xu, J., Sangthong, R., McNeil, E., Tang, R. and Chongsuvivatwong, V., 2020. Antibiotic use in chicken farms in northwestern China. Antimicrobial Resistance & Infection Control, 9(1), p.10.	China	NA				x						AH, HH
Yadav, R.K., Kaphle, H.P., Yadav, D.K., Gurung, S.C., Khatri, E. and Baral, S., 2020. Factors associated with treatment adherence among tuberculosis patients in gandaki province of Nepal. SAARC Journal of Tuberculosis, Lung Diseases and HIV/AIDS, 18(1), pp.8-21.	Nepal	MDR-TB, XDR-TB							x			HH

Zhao, H., Bian, J., Han, X., Zhang, M. and Zhan, S., 2020. Outpatient antibiotic use associated with acute upper respiratory infections in China: a nationwide cross-sectional study. International Journal of Antimicrobial Agents, 56(6), p.106193.	China	NA						x				HH
Alkhaldi, S.M., Yaseen, N.A., Bataineh, E.A., Al-Rawashdeh, B., Albadaineh, M.A., Mubarak, S.M., Jaras, R.E. and Taha, H.A., 2021. Patterns of antibiotic prescribing and appropriateness for respiratory tract infections in a teaching hospital in Jordan. *International journal of clinical practice*, *75*(6), p.e14113.	Jordon	NA						x				HH
Aslam, A., Zin, C.S., Ab Rahman, N.S., Gajdács, M., Ahmed, S.I. and Jamshed, S., 2021. Self-medication practices with antibiotics and associated factors among the public of Malaysia: a cross-sectional study. Drug, healthcare and patient safety, pp.171-181.	Malaysia	NA				x						HH
Baluku, J.B., Mukasa, D., Bongomin, F., Stadelmann, A., Nuwagira, E., Haller, S., Ntabadde, K. and Turyahabwe, S., 2021. Gender differences among patients with drug resistant tuberculosis and HIV co-infection in Uganda: a countrywide	Uganda	DR-TB	x		x							HH

retrospective cohort study. *BMC infectious diseases*, *21*(1), p.1093.												
Barasa, V. and Virhia, J., 2022. Using intersectionality to identify gendered barriers to health-seeking for febrile illness in agro-pastoralist settings in Tanzania. Frontiers in Global women's health, 2, p.746402.	Tanzania	NA			x	x						HH
Benavides, J.A., Streicker, D.G., Gonzales, M.S., Rojas-Paniagua, E. and Shiva, C., 2021. Knowledge and use of antibiotics among low-income small-scale farmers of Peru. *Preventive Veterinary Medicine*, *189*, p.105287.	Peru	NA					x					AH, HH
Blandhol, C. and Sautmann, A., 2021. Gender Differences in Children's Antibiotic Use and Adherence.	Mali	NA							x			HH
Brogdon, J.M., Sié, A., Dah, C., Ouermi, L., Coulibaly, B., Lebas, E., Zhong, L., Chen, C., Lietman, T.M., Keenan, J.D. and Doan, T., 2021. Poultry ownership and genetic antibiotic resistance determinants in the gut of preschool children. The American Journal of Tropical Medicine and Hygiene, 104(5), p.1768.	Burkina Faso	tetrecycline resistance		x								HH, AH

Chang, C.T., Lee, M., Lee, J.C.Y., Lee, N.C.T., Ng, T.Y., Shafie, A.A. and Thong, K.S., 2021. Public KAP towards COVID-19 and antibiotics resistance: a Malaysian survey of knowledge and awareness. International Journal of Environmental Research and Public Health, 18(8), p.3964.	Malaysia	COVID-19					x					HH
Cheng, Q., Xie, L., Wang, L., Lu, M., Li, Q., Wu, Y., Huang, Y., Jia, Q. and Zhao, G., 2021. Incidence density and predictors of multidrug-resistant tuberculosis among individuals with previous tuberculosis history: a 15-year retrospective cohort study. Frontiers in Public Health, 9, p.644347.	China	MDR-TB	x		x							HH
Coelho, E.C., Souza, S.B., Costa, C.C.S., Costa, L.M., Pinheiro, L.M.L., Machado, L.F.A., Silva-Oliveira, G.C., Martins, L.C., Frade, P.C.R. and Oliveira-Filho, A.B., 2021. Treponema pallidum in female sex workers from the Brazilian Marajó Archipelago: prevalence, risk factors, drug-resistant mutations and coinfections. *Transactions of the Royal Society of Tropical Medicine and Hygiene*, *115*(7), pp.792-800.	Brazil	Treponema pallidum		x								HH

Dixon, J., MacPherson, E.E., Nayiga, S., Manyau, S., Nabirye, C., Kayendeke, M., Sanudi, E., Nkaombe, A., Mareke, P., Sitole, K. and de Lima Hutchison, C., 2021. Antibiotic stories: a mixed-methods, multi-country analysis of household antibiotic use in Malawi, Uganda and Zimbabwe. BMJ Global Health, 6(11).	Malawi, Uganda, Zimbabwe	amoxicillin, cotrimoxazole and metronidazole				x						HH
Do, N.T., Vu, H.T., Nguyen, C.T., Punpuing, S., Khan, W.A., Gyapong, M., Asante, K.P., Munguambe, K., Gómez-Olivé, F.X., John-Langba, J. and Tran, T.K., 2021. Community-based antibiotic access and use in six low-income and middle-income countries: a mixed-method approach. The Lancet Global Health, 9(5), pp.e610-e619.	Mozambique, Ghana, South Africa, Bangladesh, Vietnam, Thailand	NA			x		x					HH
Santos, F.L.D., Souza, L.L.L., Bruce, A.T.I., Crispim, J.D.A., Arroyo, L.H., Ramos, A.C.V., Berra, T.Z., Alves, Y.M., Scholze, A.R., Costa, F.B.P.D. and Martoreli Junior, J.F., 2021. Patients’ perceptions regarding multidrug-resistant tuberculosis and barriers to seeking care in a priority city in Brazil during COVID-19 pandemic: a qualitative study. PloS one, 16(4), p.e0249822.	Brazil	MDR-TB		x								HH

Garbern, S.C., Chu, T.C., Yang, P., Gainey, M., Nasrin, S., Kanekar, S., Qu, K., Nelson, E.J., Leung, D.T., Ahmed, D. and Schmid, C.H., 2021. Clinical and socio-environmental determinants of multidrug-resistant Vibrio cholerae 01 in older children and adults in Bangladesh. International Journal of Infectious Diseases, 105, pp.436-441.	Bangladesh	Vibrio cholerae serogroup O1	**x**									HH
Henaine, A.M., Lahoud, N., Abdo, R., Shdeed, R., Safwan, J., Akel, M., Fahs, I., Zeenny, R., Sacre, H., Hallit, S. and Salameh, P., 2021. Knowledge of Antibiotics Use among Lebanese Adults: A study on the influence of sociodemographic characteristics. Sultan Qaboos University Medical Journal, 21(3), p.442.	Lebanon	Socio-demographic factors associated with antibiotic knowledge					x					HH
Hosoglu, S., Classen, A.Y. and Akturk, Z., 2021. Antibiotic prescription in primary care from the perspective of family physicians: a qualitative study. The Journal of Infection in Developing Countries, 15(08), pp.1117-1123.	Turkey	Antibiotic prescribing practices			x			x				HH
Ijaodola, O.A., Adeyemi, A., Bakare, R. and Salau, O.R., 2021. Determinants of HIV treatment adherence in ethnically diverse and economically disadvantaged	Nigeria	HIV							x			HH

patients in a tertiary hospital, Nigeria.												
Jiang, W., Peng, Y., Wang, X., Elbers, C., Tang, S., Huang, F., Chen, B. and Cobelens, F., 2021. Policy changes and the screening, diagnosis and treatment of drug-resistant tuberculosis patients from 2015 to 2018 in Zhejiang Province, China: a retrospective cohort study. BMJ open, 11(4), p.e047023.	China	Drug resistant tuberculosis (DR-TB)	x		x							HH
Kalam, A., Shano, S., Khan, M.A., Islam, A., Warren, N., Hassan, M.M. and Davis, M., 2021. Understanding the social drivers of antibiotic use during COVID-19 in Bangladesh: Implications for reduction of antimicrobial resistance. PLoS One, 16(12), p.e0261368.	Bangladesh	Social drivers of antibiotic use during COVID-19			**x**		x				x	HH
Kalayu, G. and Tomas, Z., 2021. Antiretroviral treatment default and associated factors among people living with HIV/AIDS in Ayder Referral Hospital, Tigray, Ethiopia. HIV & AIDS Review. International Journal of HIV-Related Problems, 20(1), pp.21-25.	Ethiopia	Antiretroviral treatment default							x			HH

Karuniawati, H., Hassali, M.A.A., Suryawati, S., Ismail, W.I., Taufik, T. and Hossain, M.S., 2021. Assessment of knowledge, attitude, and practice of antibiotic use among the population of Boyolali, Indonesia: a cross-sectional study. International journal of environmental research and public health, 18(16), p.8258.	Indonesia	KAP of antibiotic use				**x**	**x**					HH
Kaswa, M., Minga, G., Nkiere, N., Mingiedi, B., Eloko, G., Nguhiu, P. and Garcia Baena, I., 2021. The economic burden of TB-affected households in DR Congo. The International Journal of Tuberculosis and Lung Disease, 25(11), pp.923-932.	DR Congo	Tuberculosis (TB)		x	x						x	HH
Kibirige, L., Izudi, J. and Okoboi, S., 2021. Discontinuation of tuberculosis treatment among children in the Kampala Capital City Authority health facilities: a mixed-methods study. BMC infectious diseases, 21(1), p.511.	Uganda	TB			x				x	x	x	HH
Kiekens, A., Mosha, I.H., Zlatić, L., Bwire, G.M., Mangara, A., Dierckx de Casterlé, B., Decouttere, C., Vandaele, N., Sangeda, R.Z., Swalehe, O. and Cottone, P., 2021. Factors associated with HIV drug resistance in Dar ES Salaam, Tanzania: analysis of a complex	Tanzania	HIV drug resistance (HIVDR)			x				x	x	x	HH

adaptive system. Pathogens, 10(12), p.1535.												
Kizito, E., Musaazi, J., Mutesasira, K., Twinomugisha, F., Namwanje, H., Kiyemba, T., Freitas Lopez, D.B., Nicholas, N.S., Nkolo, A., Birabwa, E. and Dejene, S., 2021. Risk factors for mortality among patients diagnosed with multi-drug resistant tuberculosis in Uganda-a case-control study. *BMC infectious diseases*, *21*(1), p.292.	Uganda	MDR TB	x		x							HH
Kurowski, K.M., Marusinec, R., Amato, H.K., Saraiva-Garcia, C., Loayza, F., Salinas, L., Trueba, G. and Graham, J.P., 2021. Social and environmental determinants of community-acquired antimicrobial-resistant Escherichia coli in children living in semirural communities of Quito, Ecuador. The American journal of tropical medicine and hygiene, 105(3), p.600.	Ecuador	antimicrobial-resistant Escherichia coli		x								HH, AH, EH
Li, C., Giovenco, D., Dong, W., Smith, M.K., Golin, C.E., Fisher, E.B., Lei, G., Jiang, H., Tang, P.Y. and Muessig, K.E., 2021. Understanding How Gay, Bisexual, and Other Men Who Have Sex With Men in China Cope With HIV Care-Related Stressors.	China	HIV									x	HH

AIDS Education and Prevention, 33(1), pp.46-61.												
Li, H., Zhang, H., Xiong, J., Wang, Y., Wang, W., Wang, J., Lin, Y. and Zhang, P., 2021. Factors associated with medical follow-up adherence for patients on all-oral regimen for multidrug-resistant tuberculosis in Shenzhen, China. Patient preference and adherence, pp.1491-1496.	China	MDR-TB							x			HH
Nabirye, C., Denyer Willis, L., Nayiga, S., Kayendeke, M., Staedke, S.G. and Chandler, C.I., 2023. Antibiotic ‘entanglements’: health, labour and everyday life in an urban informal settlement in Kampala, Uganda. Critical Public Health, 33(1), pp.95-104.	Uganda	NA		x		x		x		x	x	HH,AH,EH
Numpong, S., Kengganpanich, M., Kaewkungwal, J., Pan-Ngum, W., Silachamroon, U., Kasetjaroen, Y. and Lawpoolsri, S., 2022. Confronting and coping with multidrug-resistant tuberculosis: Life experiences in Thailand. Qualitative Health Research, 32(1), pp.159-167.	Thailand	MDR TB									x	HH

Omulo, S., Lofgren, E.T., Lockwood, S., Thumbi, S.M., Bigogo, G., Ouma, A., Verani, J.R., Juma, B., Njenga, M.K., Kariuki, S. and McElwain, T.F., 2021. Carriage of antimicrobial-resistant bacteria in a high-density informal settlement in Kenya is associated with environmental risk-factors. *Antimicrobial Resistance & Infection Control*, *10*(1), p.18.	Kenya	E. coli		x		x						HH and EH
Oumer, N., Atnafu, D.D., Worku, G.T. and Tsehay, A.K., 2021. Determinants of Multi-drug resistant Tuberculosis in four treatment centers of Eastern Amhara, Ethiopia: A case-control study. The Journal of Infection in Developing Countries, 15(05), pp.687-695.	Ethiopia	Drug resistant TB/MDRTB		x	x							HH
Shadrach, B.J., Kumar, S., Deokar, K., Singh, G.V. and Goel, R., 2021. A study of multidrug resistant tuberculosis among symptomatic household contacts of MDR-TB patients. Indian Journal of Tuberculosis, 68(1), pp.25-31.	India	DR-TB	x	x								HH
Souza, L.L.L., Santos, F.L.D., Crispim, J.D.A., Fiorati, R.C., Dias, S., Bruce, A.T.I., Alves, Y.M., Ramos, A.C.V., Berra, T.Z., da Costa, F.B.P. and Alves, L.S., 2021. Causes of multidrug-resistant tuberculosis from the	Brazil	MDR-TB		x					x		x	HH

perspectives of health providers: challenges and strategies for adherence to treatment during the COVID-19 pandemic in Brazil. *BMC Health Services Research*, *21*(1), p.1033.												
Tarrant, C., Colman, A.M., Jenkins, D.R., Chattoe-Brown, E., Perera, N., Mehtar, S., Nakkawita, W.D., Bolscher, M. and Krockow, E.M., 2021. Drivers of broad-spectrum antibiotic overuse across diverse hospital contexts—A qualitative study of prescribers in the UK, Sri Lanka and South Africa. Antibiotics, 10(1), p.94.	Sri Lanka, South Africa	NA						x				HH
Theingi, P., Kamiya, Y., Myat Moe, M., Cho San, C. and Cox, S.E., 2021. Depression and its associated factors among people with multidrug-resistant tuberculosis in Myanmar. Tropical Medicine & International Health, 26(9), pp.1117-1126.	Myanmar	MDR-TB									x	HH
Timire, C., Ngwenya, M., Chirenda, J., Metcalfe, J.Z., Kranzer, K., Pedrazzoli, D., Takarinda, K.C., Nguhiu, P., Madzingaidzo, G., Ndlovu, K. and Mapuranga, T., 2021. Catastrophic costs among tuberculosis-affected households in Zimbabwe: A national health facility-based	Zimbabwe	DR-TB									x	HH

survey. Tropical Medicine & International Health, 26(10), pp.1248-1255.												
Wekunda, P.W., Aduda, D.S.O. and Guyah, B., 2021. Determinants of tuberculosis treatment interruption among patients in Vihiga County, Kenya. PLoS One, 16(12), p.e0260669.	Kenya	TB							x			HH
Wong, L.P., Alias, H., Husin, S.A., Ali, Z.B., Sim, B. and Ponnampalavanar, S.S.L.S., 2021. Factors influencing inappropriate use of antibiotics: Findings from a nationwide survey of the general public in Malaysia. PLoS One, 16(10), p.e0258698.	Malaysia	NA			x							HH
Ye, J., Zou, X., Wu, H., Chen, L., Zhou, F., Zhang, H., Kuang, H., Gong, C., Zhou, L. and Li, L., 2021. DISPARITIES IN "CATASTROPHIC" OUT-OF-POCKET COST INCURRED FROM MULTI-DRUG RESISTANT TUBERCULOSIS TREATMENT AMONG MIGRANT AND RESIDENT PATIENTS IN GUANGZHOU, GUANGDONG PROVINCE, PR CHINA. *The Southeast Asian Journal of Tropical Medicine and Public Health*, *52*(3), pp.382-402.	China	MDR-TB			x				x		x	HH

Zheng, C., Karkey, A., Wang, T., Makuka, G., van Doorn, H.R. and Lewycka, S., 2021. Determinants and patterns of antibiotic consumption for children under five in Nepal: analysis and modelling of Demographic Health Survey data from 2006 to 2016. Tropical Medicine & International Health, 26(4), pp.397-409.	Nepal	NA		x	x							HH
Abdullahi, A., Nzou, S.M., Kikuvi, G. and Mwau, M., 2022. Neisseria gonorrhoeae infection in female sex workers in an STI clinic in Nairobi, Kenya. Plos one, 17(2), p.e0263531.	Kenya	Neisseria gonorrhoeae infection		x								HH
Aslam, A., Zin, C.S., Jamshed, S., Rahman, N.S.A., Ahmed, S.I., Pallós, P. and Gajdács, M., 2022. Self-medication with antibiotics: prevalence, practices and related factors among the Pakistani public. Antibiotics, 11(6), p.795.	Pakistan	NA			x							HH
Badgeba, A., Shimbre, M.S., Gebremichael, M.A., Bogale, B., Berhanu, M. and Abdulkadir, H., 2022. Determinants of multidrug-resistant mycobacterium tuberculosis infection: a multicenter study from southern Ethiopia. Infection and Drug Resistance, pp.3523-3535.	Ethiopia	Multidrug-Resistant Mycobacterium tuberculosis	x	x					x			HH
Booth, A. and Wester, A.L., 2022. A multivariable analysis of the contribution of socioeconomic and environmental factors to	Multiple	Escherichia coli resistant to fluoroquinolones		x								HH

blood culture Escherichia coli resistant to fluoroquinolones in high-and middle-income countries. BMC Public Health, 22(1), p.354.												
Emgård, M., Mwangi, R., Mayo, C., Mshana, E., Nkini, G., Andersson, R., Lepp, M., Skovbjerg, S. and Muro, F., 2022. Antibiotic use in children under 5 years of age in Northern Tanzania: a qualitative study exploring the experiences of the caring mothers. Antimicrobial Resistance & Infection Control, 11(1), p.130.	Tanzania	Antibiotic [use] in general			x		x		x	x		HH
Essigmann, H.T., Aguilar, D.A., Perkison, W.B., Bay, K.G., Deaton, M.R., Brown, S.A., Hanis, C.L. and Brown, E.L., 2022. Epidemiology of antibiotic use and drivers of cross-border procurement in a Mexican American border community. Frontiers in Public Health, 10, p.832266.	Mexico	Choice of obtaining antibiotics		**x**	x							HH
George, S., Paranjpe, A., Nagesh, P. and Saalim, M., 2022. Barriers to treatment adherence for female tuberculosis (TB) patients during the COVID-19 pandemic: qualitative evidence from front-line TB interventions in bengaluru City, India. Indian Journal of Public Health, 66(1), pp.38-44.	India	Tuberculosis (pulmonary and extra-pulmonary during COVID-19 pandemic)							x		x	HH

Hobeika, W., Gaschet, M., Ploy, M.C., Buelow, E., Sarkis, D.K. and Dagot, C., 2022. Resistome diversity and dissemination of WHO priority antibiotic resistant pathogens in Lebanese estuaries. Antibiotics, 11(3), p.306.	Lebanon	Antibiotic resistant bacteria (ARB) and antibiotic resistance genes (ARGs)										EH
Hui, S., Chen, F., Li, Y., Cui, Y., Zhang, J., Zhang, L., Yang, Y., Liu, Y., Zhao, Y. and Lv, F., 2022. Factors associated with newly HIV infection and transmitted drug resistance among men who have sex with men in Harbin, PR China. Frontiers in Public Health, 10, p.860171.	China	HIV incidence and transmitted drug resistance (TDR)		x								HH
Jones, N., Mitchell, J., Cooke, P., Baral, S., Arjyal, A., Shrestha, A. and King, R., 2022. Gender and antimicrobial resistance: what can we learn from applying a gendered lens to data analysis using a participatory arts case study?. Frontiers in Global Women's Health, 3, p.745862.	Nepal	Gendered behaviour patterns relate to AMR			x	x	x					HH
Karimi, K.J., Ahmad, A., Duse, A., Mwanthi, M. and Ayah, R., 2022. Prevalence of antibiotic use and disposal at household level in informal settlements of Kisumu, Kenya. *International Journal of Environmental Research and Public Health*, *20*(1), p.287.	Kenya	Antibiotic use and disposal at household level		x			x					HH

Khine Zaw, Y., Bawk, J.S. and De Lima Hutchison, C., 2022. Negotiating authoritarian law and (dis) order: Medicines, drug shops, and regulators in a poor Yangon suburb. Critical Public Health, 32(5), pp.641-653.	Myanmar	People's views on medicine sales, access, and regulation, how these work in practice, and whether they align with Myanmar's drug laws and AMR NAP										HH
Ladha, N., Bhardwaj, P., Chauhan, N.K., Naveen, K.H.S., Nag, V.L. and Giribabu, D., 2022. Determinants, risk factors and spatial analysis of multi-drug resistant pulmonary tuberculosis in Jodhpur, India. Monaldi Archives for Chest Disease, 92(4).	India	MDR-TB	x	x								HH
Li, X., Pang, X. and Zhang, F., 2022. Evaluation of Mobile Application for the Management of Tuberculosis Patients in Tianjin During 2019–2020. *Patient preference and adherence*, pp.321-329.	China	TB							x		x	HH
Makabayi-Mugabe, R., Musaazi, J., Zawedde-Muyanja, S., Kizito, E., Namwanje, H., Aleu, P., Charlet, D., Freitas Lopez, D.B., Brightman, H., Turyahabwe, S. and Nkolo, A., 2022. Developing a patient-centered community-based model for management of multi-drug resistant tuberculosis	Uganda	MDR-TB			x					x	x	HH

in Uganda: a discrete choice experiment. BMC health services research, 22(1), p.154.												
Malik, A.A., Khan, U., Khan, P., Anwar, A., Salahuddin, N., Khowaja, S., Khan, A.J., Khan, S., Hussain, H. and Amanullah, F., 2022. Drug-resistant tuberculosis treatment outcomes among children and adolescents in Karachi, Pakistan. Tropical medicine and infectious disease, 7(12), p.418.	Pakistan	DR-TB		x							x	HH
Miyano, S., Htoon, T.T., Nozaki, I., Pe, E.H. and Tin, H.H., 2022. Public knowledge, practices, and awareness of antibiotics and antibiotic resistance in Myanmar: The first national mobile phone panel survey. PLoS One, 17(8), p.e0273380.	Myanmar	NA				x		x	x			HH
Musema, G.M.A., Akilimali, P.Z., Za Balega, T.K.N., Tshala-Katumbay, D. and Lusamba, P.S.D., 2022. Predictive factors of HIV-1 drug resistance and its distribution among female sex workers in the Democratic Republic of the Congo (DRC). International journal of environmental research and public health, 19(4), p.2021.	DRC	HIV		x								HH

Ndaki, Pendo M., Martha F. Mushi, Joseph R. Mwanga, Eveline T. Konje, Stella Mugassa, Msilikale W. Manyiri, Stanley M. Mwita et al. "Non-prescribed antibiotic dispensing practices for symptoms of urinary tract infection in community pharmacies and accredited drug dispensing outlets in Tanzania: a simulated clients approach." BMC Primary Care 23, no. 1 (2022): 287.	Tanzania	UTI						x				HH
Nyeko, R., Otim, F., Obiya, E.M. and Abala, C., 2022. Pre-hospital exposures to antibiotics among children presenting with fever in northern Uganda: a facility-based cross-sectional study. BMC pediatrics, 22(1), p.322.	Uganda	fever			x	x		x				HH
Poompruek, P., Perris, A., Whanpuch, P., Chandler, C.I. and Sringernyuang, L., 2022. Mystifying medicines and maximising profit: Antibiotic distribution in community pharmacies in Thailand. Global Public Health, 17(12), pp.3931-3943.	Thailand	NA										HH
Redwood, L., Fox, G.J., Nguyen, T.A., Bernarys, S., Mason, P., Vu, V.A., Nguyen, V.N. and Mitchell, E.M., 2022. Good citizens, perfect patients, and family reputation: Stigma and prolonged isolation in people with drug-resistant	Vietnam	DR-TB									x	HH

tuberculosis in Vietnam. *PLOS Global Public Health*, *2*(6), p.e0000681.												
Sharma, R., Bakshi, H., Prajapati, S., Bhatt, G.S., Mehta, R., Rami, K.C., Mehta, P., Shah, T., Dave, R. and Peerzada, A., 2022. Prevalence and determinants of depression among multi drug resistant (MDR) TB cases registered under National Tuberculosis Elimination Program in Ahmedabad City. Indian Journal of Community Medicine, 47(1), pp.45-49.	India	MDR-TB									x	HH
Shembo, A.K.P., Musumari, P.M., Srithanaviboonchai, K., Tangmunkongvorakul, A. and Dalleur, O., 2022. A qualitative study on community use of antibiotics in Kinshasa, Democratic Republic of Congo. PLoS One, 17(4), p.e0267544.	DRC	NA		x	x							HH
Shukla, V., Ray, K., Dutta, S. and Basu, M., 2022. Self-medication practices and health seeking behaviour among residents of selected villages in a block of West Bengal: a mixed-methods study.	India	NA			x							HH

Soundararajan, S.S., Pavithra, G.B., Preeti, P., Nisha, B., Vengadassalapathy, S., Dutta, R. and Parasuraman, G., 2022. Social determinants and risk factors associated with multi-drug resistant tuberculosis among tuberculosis patients in Tamilnadu-a case-control study.	India	MDR-TB		x								HH
Taylor, H.A., Dowdy, D.W., Searle, A.R., Stennett, A.L., Dukhanin, V., Zwerling, A.A. and Merritt, M.W., 2022. Disadvantage and the experience of treatment for multidrug-resistant tuberculosis (MDR-TB). SSM-Qualitative Research in Health, 2, p.100042.	South Africa, Uganda	MDR-TB			x				x	x	x	HH
Yuan, D., Liu, Y., Zhou, Y., Shi, L., Chen, J., Lu, J., Fu, G. and Wang, B., 2022. Men who have sex with men is the high-risk drug resistance population: A meta-analysis of HIV-1 drug resistance profiles and trends in China. *Journal of Clinical Pharmacy and Therapeutics*, *47*(11), pp.1729-1737.	China	HIV		x								HH
Yunita, S.L., Yang, H.W., Chen, Y.C., Kao, L.T., Lu, Y.Z., Wen, Y.L., To, S.Y. and Huang, Y.L., 2022. Knowledge and practices related to antibiotic use among women in Malang, Indonesia. Frontiers in pharmacology, 13, p.1019303.	Indonesia	NA			x	x			x			HH

Rajendran, M., Aghamohammadi, N., Ahmad Zaki, R. and Abu Bakar, Z., Prevalence and Associated Risk Factors of Multidrug Resistant Tuberculosis in Malaysia. Available at SSRN 4141364.	Malaysia	MDR-TB			x				x			HH
Allel, K., Labarca, J., Carvajal, C., Garcia, P., Cifuentes, M., Silva, F., Munita, J.M. and Undurraga, E.A., 2023. Trends and socioeconomic, demographic, and environmental factors associated with antimicrobial resistance: a longitudinal analysis in 39 hospitals in Chile 2008–2017. *The Lancet Regional Health–Americas*, *21*.	Chile	Klebsiella pneumoniae resistant to third-generation cephalosporins and carbapenems, and vancomycin-resistant Enterococcus faecium		x				x				HH
Arushothy, R., Ali, M.R.M., Zambri, H.F., Muthu, V., Hashim, R., Chieng, S. and Nathan, S., 2024. Assessing the national antibiotic surveillance data to identify burden for melioidosis in Malaysia. IJID regions, 10, pp.94-99.	Malaysia	B. pseudomallei and melioidosis	x	x	x							HH, EH
Campbell, Z.A., Njiru, N., Mhone, A.L., Makumi, A., Moineau, S. and Svitek, N., 2023. Gender-responsive design of bacteriophage products to enhance adoption by chicken keepers in Kenya. Viruses, 15(3), p.746.	Kenya	Bacteriophage Products			x							AH

Chand, K., Butt, M.I. and Tahir, H.M., 2023. Parental attitude, knowledge, and practices regarding the usage of antibiotics for upper respiratory tract infections in children during the COVID-19 pandemic. *Cureus*, *15*(6).	India	Upper respiratory tract infections					x					HH
Medeiros, Regielle Luiza de, Adriana da Silva Rezende Moreira, Ana Carolina de Oliveira Jeronymo Neves, Viviane de Jesus Leite, Isabela Neves de Almeida, Fernanda Carvalho de Queiroz Mello, and Afrânio Kritski. "Analysis of catastrophic costs incurred by patients with multidrug-resistant tuberculosis in an outpatient clinic in the state of Rio de Janeiro." Revista da Sociedade Brasileira de Medicina Tropical 56 (2023): e0148-2023.	Brazil	MDR-TB									x	HH
Fu, M., Gong, Z., Zhu, Y., Li, C., Zhou, Y., Hu, L., Li, H., Wushouer, H., Guan, X. and Shi, L., 2023. Inappropriate antibiotic prescribing in primary healthcare facilities in China: a nationwide survey, 2017–2019. Clinical Microbiology and Infection, 29(5), pp.602-609.	China	Antibiotic [prescriptions]						x				HH

Fuhrmeister, E.R., Harvey, A.P., Nadimpalli, M.L., Gallandat, K., Ambelu, A., Arnold, B.F., Brown, J., Cumming, O., Earl, A.M., Kang, G. and Kariuki, S., 2023. Evaluating the relationship between community water and sanitation access and the global burden of antibiotic resistance: an ecological study. The Lancet Microbe, 4(8), pp.e591-e600.	Multi-country (low-income, lower-middle income, and upper-middle income countries_	Antibiotic resistance genes (ARGs)		x								EH
Green, D.L., Keenan, K., Fredricks, K.J., Huque, S.I., Mushi, M.F., Kansiime, C., Asiimwe, B., Kiiru, J., Mshana, S.E., Neema, S. and Mwanga, J.R., 2023. The role of multidimensional poverty in antibiotic misuse: a mixed-methods study of self-medication and non-adherence in Kenya, Tanzania, and Uganda. *The Lancet Global Health*, *11*(1), pp.e59-e68.	Kenya, Tanzania, and Uganda	Urinary Tract Infection (UTI) symptoms			x				**x**	x		HH
Gyamerah, Akua O., Ezra Kinzer, Gloria Aidoo-Frimpong, Guro Sorensen, Matilda D. Mensah, Kelly D. Taylor, Naa Ashiley Vanderpuye, and Sheri A. Lippman. "PrEP knowledge, acceptability, and implementation in Ghana: perspectives of HIV service providers and MSM, trans women, and gender diverse individuals living with HIV." *PLOS*	Ghana	HIV (ARV drug use and resistance)	x	x	x						x	HH

global public health 3, no. 6 (2023): e0001956.												
Haenssgen, M.J., Charoenboon, N., Early, A. and Althaus, T., 2023. Community-level incidence and treatment seeking during febrile illness: Insights from health behaviour surveys in rural Thailand and Laos. Tropical Medicine & International Health, 28(10), pp.806-816.	Thailand, Laos	Febrile illness			x			x				HH
Hibbard, R., Chapot, L., Yusuf, H., Ariyanto, K.B., Maulana, K.Y., Febriyani, W., Cameron, A., Vergne, T., Faverjon, C. and Paul, M.C., 2023. “It’sa habit. They’ve been doing it for decades and they feel good and safe.”: A qualitative study of barriers and opportunities to changing antimicrobial use in the Indonesian poultry sector. *Plos one*, *18*(9), p.e0291556.	Indonesia	Behavioural change in AMU in poultry sector										HH, AH
Hordofa, G., Mulatu, G. and Daka, D., 2023. Prevalence, drug-susceptibility pattern and associated factors of Mycobacterium tuberculosis infection among prisoners in western Arsi zonal prisons,	Ethiopia	Pulmonary tuberculosis (PTB)	x				x					HH

Oromia, South West Ethiopia. *IJID regions*, *9*, pp.1-6.												
Hordofa, G., Mulatu, G. and Daka, D., 2023. Prevalence, drug-susceptibility pattern and associated factors of Mycobacterium tuberculosis infection among prisoners in western Arsi zonal prisons, Oromia, South West Ethiopia. *IJID regions*, *9*, pp.1-6.	Kenya	Contamination of groundwater with sulfamethoxazole and antibiotic resistant Escherichia coli		x								EH
Kayendeke, M., Denyer-Willis, L., Nayiga, S., Nabirye, C., Fortané, N., Staedke, S.G. and Chandler, C.I., 2023. Pharmaceuticalised livelihoods: antibiotics and the rise of 'quick farming'in peri-urban Uganda. *Journal of biosocial science*, *55*(6), pp.995-1014.	Uganda	Quick farming phenomenon				x						HH, AH
Kim, S., Hesseling, A.C., Wu, X., Hughes, M.D., Shah, N.S., Gaikwad, S., Kumarasamy, N., Mitchell, E., Leon, M., Gonzales, P. and Badal-Faesen, S., 2023. Factors associated with prevalent Mycobacterium tuberculosis infection and disease among adolescents and adults exposed to rifampin-resistant tuberculosis in the household. *PLoS One*, *18*(3), p.e0283290.	Multiple countries	Mycobacterium tuberculosis infection and disease	x	x								HH

Kukula, V.A., Odopey, S., Arthur, E., Odonkor, G., Awini, E., Adjei, A., Salami, O., Nkeramahame, J., Horgan, P., Olliaro, P. and Williams, J., 2023. Understanding health worker and community antibiotic prescription-adherence practices for acute febrile illness: a nested qualitative study in the shai-osudoku district of ghana and the development of a training-and-communication intervention. Clinical Infectious Diseases, 77(Supplement_2), pp.S182-S190.	Ghana	Accute febrile illness						x	x		x	HH
Lu, J., Xu, Y., Li, Z., Chen, X., Lin, H. and Zhao, Q., 2023. Diagnosis and treatment pathway of MDR/RR-TB in Taizhou, Zhejiang Province, China. Tropical medicine and infectious disease, 8(2), p.79.	China	MDR/RR-TB		x	x				x			HH
Mete, B., Salcan, T. and Demirhindi, H., 2023. Behaviour and perception of parents on irrational use of antibiotics in children at primary care level: A cross-sectional study from Turkey. Asian Pacific Journal of Tropical Medicine, 16(6), pp.253-260.	Turkey	NA				x	x	x	x			HH
Musoke, D., Lubega, G.B., Obeng, M.B., Brandish, C., Winter, J., Niyongabo, F., Russell-Hobbs, K., Ng, B.Y., Mugisha, L., Amir, S. and Kitutu, F.E., 2023. Knowledge,	Uganda	NA					x		x			HH,AH

perceptions and practices on antimicrobial resistance in humans and animals in Wakiso district, Uganda: A cross sectional study. PLOS Global Public Health, 3(12), p.e0002701.												
Lwembe, R.M., Knight, V., Randa, D., Śнамciborski, R.J. and Ayodo, G., 2023. Association of ART non-adherence with self-reported behavior and lifestyle among injecting drug users in the coastal region of Kenya. Scientific African, 20, p.e01598.	Kenya	HIV		x							x	HH
Oladimeji, O., Atiba, B.P., Anyiam, F.E., Odugbemi, B.A., Afolaranmi, T., Zoakah, A.I. and Horsburgh, C.R., 2023. Gender and drug-resistant tuberculosis in Nigeria. Tropical Medicine and Infectious Disease, 8(2), p.104.	Nigeria	Drug resistant TB/MDRTB	x	x							x	HH
Ong'era, E., Kagira, J., Maina, N., Kiboi, D., Waititu, K., Michira, L. and Ngotho, M., 2023. Prevalence and Potential Risk Factors for the Acquisition of Antibiotic-Resistant Staphylococcus spp. Bacteria Among Pastoralist Farmers in Kajiado Central Subcounty, Kenya. *BioMed Research International*, *2023*(1), p.3573056.	Kenya	Staphylococcus spp. bacteria		x								HH and AH

Oumer, A., Ale, A., Hamza, A. and Dagne, I., 2023. Extent and Correlates of Self-Medication Practice among Community-Dwelling Adults in Eastern Ethiopia. BioMed Research International, 2023(1), p.4726010.	Ethiopia	MDRTB	x		x	x						HH
Pham, T.A.M., Forse, R., Codlin, A.J., Phan, T.H.Y., Nguyen, T.T., Nguyen, N., Vo, L.N.Q., Dat, P.T., Minh, H.D.T., Nguyen, L.H. and Nguyen, H.B., 2023. Determinants of catastrophic costs among households affected by multi-drug resistant tuberculosis in Ho Chi Minh City, Viet Nam: a prospective cohort study. *BMC Public Health*, *23*(1), p.2372.	Vietnam	MDR-TB									x	HH
Rousham, E.K., Nahar, P., Uddin, M.R., Islam, M.A., Nizame, F.A., Khisa, N., Akter, S.S., Munim, M.S., Rahman, M. and Unicomb, L., 2023. Gender and urban-rural influences on antibiotic purchasing and prescription use in retail drug shops: a one health study. BMC Public Health, 23(1), p.229.	Bangladesh	NA			x							HH
Sitotaw, B. and Philipos, W., 2023. Knowledge, Attitude, and Practices (KAP) on Antibiotic Use and Disposal Ways in Sidama Region, Ethiopia: A Community-Based Cross-Sectional Survey.	Ethiopia	NA					x					HH

The Scientific World Journal, 2023(1), p.8774634.												
Subedi, D., Jyoti, S., Thapa, B., Paudel, S., Shrestha, P., Sapkota, D., Bhatt, B.R., Adhikari, H., Poudel, U., Gautam, A. and Nepal, R., 2023. Knowledge, attitude, and practice of antibiotic use and resistance among poultry farmers in Nepal. Antibiotics, 12(9), p.1369.	Nepal	NA		x			x					HH, AH
Timire, C., Kranzer, K., Pedrazzoli, D., Kavenga, F., Kasozi, S., Mbiba, F. and Bond, V., 2023. Coping with drug resistant tuberculosis alongside COVID-19 and other stressors in Zimbabwe: A qualitative study. PLOS Global Public Health, 3(8), p.e0001706.	Zimbabwe	DR-TB			x						x	HH
Wakjira, M.K., Sandy, P.T. and Mavhandu-Mudzusi, A.H., 2023. Patients' perceived quality of care and their satisfaction with care given for MDR-TB at referral hospitals in Ethiopia. *Plos one*, *18*(2), p.e0270439.	Ethiopia	MDR-TB								x	x	HH
Abubakar, M., Ullah, M., Shaheen, M.A. and Abdullah, O., 2024. Why do patients with DR-TB do not complete their treatment? Findings of a qualitative study from Pakistan. BMJ Open Respiratory Research, 11(1).	Pakistan	DR-TB							x			HH

Afari-Asiedu, S., Oppong, F.B., Tostmann, A., Ali Abdulai, M., Boamah-Kaali, E., Gyaase, S., Agyei, O., Kinsman, J., Hulscher, M., Wertheim, H.F. and Asante, K.P., 2020. Determinants of inappropriate antibiotics use in rural central Ghana using a mixed methods approach. Frontiers in Public Health, 8, p.90.	Ghana	NA		x	x			x	x			HH
Akalu, T.Y., Clements, A.C., Gebreyohannes, E.A., Xu, Z., Bai, L. and Alene, K.A., 2024. Risk factors for diagnosis and treatment delay among patients with multidrug-resistant tuberculosis in Hunan Province, China. *BMC Infectious Diseases*, *24*(1), p.159.	China	MDR-TB			x							HH
Akalu, T.Y., Clements, A.C., Xu, Z., Bai, L. and Alene, K.A., 2024. Determinants of drug-resistant tuberculosis in Hunan province, China: a case-control study. BMC Infectious Diseases, 24(1), p.198.	China	DR-TB			x							HH
Al Kady, C., Moussally, K., Chreif, W., Farra, A., Caluwaerts, S., Wertheim, H., Soukarieh, D., Gomez, F.G., Dibiasi, J. and Lenglet, A., 2024. Overuse of antibiotics for urinary tract infections in pregnant refugees, Lebanon. Bulletin of the World Health Organization, 102(6), p.389.	Lebanon	NA						x				HH

Barasa, V., 2024. A one health approach to tackling AMR and why gender matters: findings from pastoralist communities in Tanzania. *Frontiers in global women's health*, *5*, p.1429203.	Tanzania	NA		x	x	x						HH, AH
Cuboia, N., Mutaquiha, C., Manhiça, I., José, B., Amaro, M., Pfumo-Cuboia, I., Nitrogénio, L., Reis-Pardal, J., Zindoga, P., Couto, A. and Azevedo, L., 2024. Spatial distribution and predictors of drug-resistant tuberculosis incidence in Mozambique: A nationwide Bayesian disease mapping study. Tropical Medicine & International Health, 29(12), pp.1051-1061.	Mozambique	DR-TB	x	x	x							HH
Edessa, D., Kumsa, F.A., Dinsa, G. and Oljira, L., 2024. Drug providers' perspectives on antibiotic misuse practices in eastern Ethiopia: a qualitative study. BMJ open, 14(8), p.e085352.	Ethiopia	NA				x		x				HH
Gautham, M., Bhattacharyya, S., Maity, S., Roy, M.B., Balasubramaniam, P., Ebata, A. and Bloom, G., 2024. "Just as curry is needed to eat rice, antibiotics are needed to cure fever"—a qualitative study of individual, community and health system-level influences on community antibiotic practices in	India	Antibiotic [use/practices] in the community			x		x	x	x			HH

rural West Bengal, India. BMJ open, 14(2), p.e076616.												
González-Villoria, A.M., García Quiroz, A.D., Osorio Guzmán, E.U., Suarez-Herrera, J.C. and Abeldaño Zuñiga, R.A., 2024, January. Knowledge, attitudes, and practices of parents in the use of antibiotics: a case study in a Mexican indigenous community. In Healthcare (Vol. 12, No. 3, p. 294). MDPI.	Mexico	Knowledge, attitudes, and practices (KAP) regarding antibiotic use	x	x								HH
Hackman, H.K., Annison, L., Arhin, R.E., Adjei, G.O., Otu, P., Arthur-Hayford, E., Annison, S. and Borteih, B.B., 2024. Self-medication with antibiotics during the COVID-19 pandemic: A cross-sectional study among adults in Tema, Ghana. *Plos one*, *19*(6), p.e0305602.	Ghana	Antibiotic self-medication				x						HH
Hanh, N.T.H., Tram, P.T.P., Ha, H.T.T., Duc, B.H., Huong, P.T.T., Quoc, N.C., Thanh, D.C., Hien, B.T., Ha, N.T.T., Mai, L.T.Q. and Anh, D.D., 2022. Detection of antiretroviral drug-resistant mutations and HIV-1 subtypes in circulation among men who have sex with men, SEM females and female sex workers: results of Vietnam's HIV Sentinel Surveillance Plus (HSS+) system,	Vietnam	Antiretroviral Drug-Resistant Mutations and HIV-1 Subtypes		x								HH

2018–2020. *JAIDS Journal of Acquired Immune Deficiency Syndromes*, pp.10-1097.												
Hosu, M.C., Faye, L.M. and Apalata, T., 2024. Comorbidities and Treatment Outcomes in Patients Diagnosed with Drug-Resistant Tuberculosis in Rural Eastern Cape Province, South Africa. Diseases, 12(11), p.296.	South Africa	Drug-resistant tuberculosis (DR-TB)	x						x			HH
Jarab, A.S., Al-Alawneh, T.O., Alshogran, O.Y., Heshmeh, S.A., Mukattash, T.L., Naser, Y.A. and Alefishat, E., 2024. Knowledge and attitude of healthcare prescribers and pharmacists toward antimicrobial stewardship program and the barriers for its implementation. Antimicrobial Resistance & Infection Control, 13(1), p.35.	Jordon	Antimicrobial stewardship programme					x					HH
Hesari, D.K., Aljadeeah, S., Brhlikova, P., Hyzam, D., Komakech, H., Rueda, J.S.P., Cañas, J.O., Ching, C., Orubu, S., Acevedo, O.B. and Basaleem, H., 2024. Access to and utilisation of antimicrobials among forcibly displaced persons in Uganda, Yemen and Colombia: a pilot	Uganda, Yemen, Colombia	Access to and utilisation of antimicrobials		x	x	x				x		HH

cross-sectional survey. BMJ open, 14(7), p.e084734.												
Kanakaraj, R., Umamaheswari, R. and Subramaniam, S., 2024. Depression and its role in adherence to anti-retroviral treatment in people living with HIV and AIDS in Chennai. *Journal of Family Medicine and Primary Care*, *13*(4), pp.1507-1510.	India	Anti-retroviral treatment (ART)							x		x	HH
Keenan, K., Papathomas, M., Mshana, S.E., Asiimwe, B., Kiiru, J., Lynch, A.G., Kesby, M., Neema, S., Mwanga, J.R., Mushi, M.F. and Jing, W., 2024. Intersecting social and environmental determinants of multidrug-resistant urinary tract infections in East Africa beyond antibiotic use. Nature Communications, 15(1), p.9418.	Kenya, Tanzania, Uganda	Risk of multi-drug resistant urinary tract infections (MDR UTIs)		**x**								HH
Kumar, C.K., Gleason, A.C., Parameswaran, G.G., Summan, A., Klein, E., Laxminarayan, R. and Nandi, A., 2024. Routine immunization against Streptococcus pneumoniae and Haemophilus influenzae type B and antibiotic consumption in India: a dynamic modeling analysis. The Lancet Regional Health-Southeast Asia, 31.	India	Streptococcus pneumoniae and Haemophilus influenzae type B										HH

Madaki, S., Mohammed, Y., Rogo, L.D., Yusuf, M. and Bala, Y.G., 2024. Age and gender in drug resistance tuberculosis: A cross-sectional case study at a national tuberculosis reference hospital in Nigeria. *Journal of Global Antimicrobial Resistance*, *39*, pp.175-183.	Nigeria	DR-TB	x	x					x	x		HH
Mahbub, T., Mathur, T., Isaakidis, P. and Daftary, A., 2024. "One-by-one, TB took everything away from me": A photovoice exploration of stigma in women with drug-resistant tuberculosis in Mumbai. *Affilia*, *39*(1), pp.148-169.	India	DR-TB									x	HH
Manikam, L., Faijue, D.D., Shringarpure, K., Sheth, M., Factor-Livak, P., Parikh, P., Altamirano-Medina, H., Aisyah, D.N., Sharma, R., Chaturvedi, H. and Sarkar, K., 2024. Understanding one health challenges in marginalized urban settings: A patient and public involvement (PPI) approach from the CHIP consortium activities across four global cities. *One Health*, *19*, p.100919.	India, Indonesia, Chile, and Turkey		x		x	x						HH, AH, EH
Mannan, A., Chakma, K., Dewan, G., Saha, A., Chy, N.U.H.A., Mehedi, H.H., Hossain, A., Wnaiza, J., Ahsan, M.T., Rana, M.M. and Alam, N., 2024. Prevalence and determinants of	Bangladesh	NA				x	x					HH

antibiotics self-medication among indigenous people of Bangladesh: a cross-sectional study. *BMJ open*, *14*(3), p.e071504.												
Matakone, M., Founou, R.C., Founou, L.L., Dimani, B.D., Koudoum, P.L., Fonkoua, M.C., Boum-Ii, Y., Gonsu, H. and Noubom, M., 2024. Multi-drug resistant (MDR) and extended-spectrum β-lactamase (ESBL) producing Escherichia coli isolated from slaughtered pigs and slaughterhouse workers in Yaoundé, Cameroon. *One Health*, *19*, p.100885.	Cameroon	Multi-drug resistant (MDR) and extended-spectrum β-lactamase (ESBL) producing Escherichia coli	x	x								HH,AH,EH
Muhummed, A.M., Alemu, A., Maidane, Y.O., Tschopp, R., Hattendorf, J., Vonaesch, P., Zinsstag, J. and Cissé, G., 2024. Knowledge, attitudes, and practices of rural communities regarding antimicrobial resistance and climate change in Adadle District, Somali Region, Ethiopia: a mixed-methods study. *Antibiotics*, *13*(4), p.292.	Ethiopia	NA				x		x				HH,AH,EH
Mukherjee, A., Surial, R., Sahay, S., Thakral, Y. and Gondara, A., 2024. Social and cultural determinants of antibiotics prescriptions: analysis from a public community health centre in North India. *Frontiers in Pharmacology*, *15*, p.1277628.	India	NA					x	x	x			HH

Murugan, Y., Patel, N., Kumar, V., Gandhi, R., Patel, N. and Bhai, V.K., 2024. Mental Health Impacts of Multidrug-Resistant Tuberculosis in Patients and Household Contacts: A Mixed Methods Study. *Cureus*, *16*(5).	India	MDR TB									x	HH
Nguyen, Y.H.T., van Doorn, R., Van Nuil, J.I. and Lewycka, S., 2024. Dilemmas of care: Healthcare seeking behaviours and antibiotic use among women in rural communities in Nam Dinh Province, Vietnam. *Social Science & Medicine*, *363*, p.117483.	Vietnam	NA				x						HH and EH
Pauzi, Z.M., Hassan, B.A.R., Neo, C.F., Mohammed, A.H., Blebil, A. and Dujaili, J., 2024. Antibiotic use and resistance in a tertiary care hospital: knowledge and attitude among patients of orthopaedic and surgical wards in Malaysia. *Journal of Pharmaceutical Health Services Research*, *15*(1), p.rmab068.	Malaysia	NA					x					HH
Ronse, M., Nguyen, T.T., Nguyen, X.X., Ingelbeen, B., Schneiders, M.L., Tran, D.T., Muela Ribera, J., Gryseels, C. and Peeters Grietens, K., 2024. Use of antimicrobials and other medical products in an ethnic minority context of South-Central Vietnam: A qualitative study of	Vietnam	NA			x							HH

vulnerability. *PLOS Global Public Health*, *4*(4), p.e0002982.												
Shah, V., Yogesh, M., Kothari, D.R., Gandhi, R.B. and Nagda, J.J., 2024. Audit of risk factors of drug-sensitive, drug-resistant tuberculosis disease, a case-control study of patients registered under NTEP, Gujarat. *Journal of Family Medicine and Primary Care*, *13*(9), pp.3614-3620.	India	DR-TB	x	x								HH
