# Supplementary material for: Antimicrobial resistance, equity and justice in low- and middle-income countries: an intersectional critical interpretive synthesis
Source: Nat Commun. 2025 Oct 13;16:9078. doi: 10.1038/s41467-025-64137-z (PMC12518637; doi:10.1038/s41467-025-64137-z)
Supplement: Supplementary file 2 — Reporting Summary [file 41467_2025_64137_MOESM2_ESM.pdf]

nature portfolio

Corresponding author(s): Katy Davis

Last updated by author(s): Jul 10, 2025

Reporting Summary

Nature Portfolio wishes to improve the reproducibility of the work that we publish. This form provides structure for consistency and transparency in reporting. For further information on Nature Portfolio policies, see our Editorial Policies and the Editorial Policy Checklist.

Statistics

For all statistical analyses, confirm that the following items are present in the figure legend, table legend, main text, or Methods section.

n/a	Confirmed	
☐	☒	The exact sample size (*n*) for each experimental group/condition, given as a discrete number and unit of measurement
☒	☐	A statement on whether measurements were taken from distinct samples or whether the same sample was measured repeatedly
☒	☐	The statistical test(s) used AND whether they are one- or two-sided<br>*Only common tests should be described solely by name; describe more complex techniques in the Methods section.*
☒	☐	A description of all covariates tested
☒	☐	A description of any assumptions or corrections, such as tests of normality and adjustment for multiple comparisons
☐	☒	A full description of the statistical parameters including central tendency (e.g. means) or other basic estimates (e.g. regression coefficient) AND variation (e.g. standard deviation) or associated estimates of uncertainty (e.g. confidence intervals)
☒	☐	For null hypothesis testing, the test statistic (e.g. *F*, *t*, *r*) with confidence intervals, effect sizes, degrees of freedom and *P* value noted<br>*Give P values as exact values whenever suitable.*
☒	☐	For Bayesian analysis, information on the choice of priors and Markov chain Monte Carlo settings
☒	☐	For hierarchical and complex designs, identification of the appropriate level for tests and full reporting of outcomes
☒	☐	Estimates of effect sizes (e.g. Cohen's *d*, Pearson's *r*), indicating how they were calculated

Our web collection on statistics for biologists contains articles on many of the points above.

Software and code

Policy information about availability of computer code

Data collection	Screening: Microsoft Excel Version 16.98 and Rayyan (online, free version). Data extraction: Microsoft Excel Version 16.98
Data analysis	Microsoft Excel Version 16.98, QGIS (data presentation)

For manuscripts utilizing custom algorithms or software that are central to the research but not yet described in published literature, software must be made available to editors and reviewers. We strongly encourage code deposition in a community repository (e.g. GitHub). See the Nature Portfolio guidelines for submitting code & software for further information.

Data

Policy information about availability of data

All manuscripts must include a data availability statement. This statement should provide the following information, where applicable:

- Accession codes, unique identifiers, or web links for publicly available datasets
- A description of any restrictions on data availability
- For clinical datasets or third party data, please ensure that the statement adheres to our policy

Data included in this article is sourced from publicly available peer-reviewed journal articles. We have included a list of included articles alongside our analysis decisions in the supplementary materials as Table S4: Included articles.

Research involving human participants, their data, or biological material

Policy information about studies with human participants or human data. See also policy information about sex, gender (identity/presentation), and sexual orientation and race, ethnicity and racism.

Reporting on sex and gender	This research did not include human participants
Reporting on race, ethnicity, or other socially relevant groupings	This research did not include human participants
Population characteristics	This research did not include human participants
Recruitment	This research did not include human participants
Ethics oversight	This research did not include human participants

Note that full information on the approval of the study protocol must also be provided in the manuscript.

Field-specific reporting

Please select the one below that is the best fit for your research. If you are not sure, read the appropriate sections before making your selection.

☐ Life sciences ☒ Behavioural & social sciences ☐ Ecological, evolutionary & environmental sciences

For a reference copy of the document with all sections, see nature.com/documents/nr-reporting-summary-flat.pdf

Behavioural & social sciences study design

All studies must disclose on these points even when the disclosure is negative.

Study description	This manuscript reports the results of a critical interpretive analysis of peer reviewed literature, which includes mixed methods approaches, drawing primarily upon qualitative methods (content analysis)
Research sample	181 peer reviewed journal articles from the period 2019-2024
Sampling strategy	Database searches: Medline, Scopus, Global Health
Data collection	Microsoft excel was used to collect and store results of database searches. As per literature review methods, we reviewed all peer reviewed journal articles that were returned in our database search and met our inclusion criteria.
Timing	Peer-reviewed literature published in the period 2019-2024 was collected. Database searches were run in December 2024
Data exclusions	Exclusion criteria include: Articles that reported only on burden of disease and did not explore reasons for identified trends, Articles that focus on high income countries, Articles published before 2014, Commentaries, review articles.
Non-participation	This research did not include human participants
Randomization	This research did not include human participants

Reporting for specific materials, systems and methods

We require information from authors about some types of materials, experimental systems and methods used in many studies. Here, indicate whether each material, system or method listed is relevant to your study. If you are not sure if a list item applies to your research, read the appropriate section before selecting a response.

Materials & experimental systems

n/a	Involved in the study
☒	☐ Antibodies
☒	☐ Eukaryotic cell lines
☒	☐ Palaeontology and archaeology
☒	☐ Animals and other organisms
☒	☐ Clinical data
☒	☐ Dual use research of concern
☒	☐ Plants

Methods

n/a	Involved in the study
☒	☐ ChIP-seq
☒	☐ Flow cytometry
☒	☐ MRI-based neuroimaging

Plants

Seed stocks

This research did not include plant specimens

Novel plant genotypes

This research did not include plant specimens

Authentication

This research did not include plant specimens
